# Functional diversity of home gardens and their agrobiodiversity conservation benefits in Benin, West Africa

**DOI:** 10.1186/s13002-017-0192-5

**Published:** 2017-11-25

**Authors:** Rodrigue Castro Gbedomon, Valère Kolawolé Salako, Adandé Belarmain Fandohan, Alix Frank Rodrigue Idohou, Romain Glèlè Kakaї, Achille Ephrem Assogbadjo

**Affiliations:** 1 Laboratoire de Biomathématiques et d’Estimations Forestières, Faculté des Sciences Agronomiques, Université d’Abomey-Calavi, 04 BP 1525, Cotonou, Benin; 2 Laboratoire d’Ecologie Appliquée, Faculté des Sciences Agronomiques, Université d’Abomey-Calavi, 01 BP 526, Cotonou, Benin; 3 Ecole de Foresterie et d’Ingénierie du Bois, Université Nationale d’Agriculture, Porto Novo 01 BP 5996, Cotonou, Benin

**Keywords:** Function, Home gardens, Agrobiodiversity, Clustering, Crop wild relatives, Crops, Wild plant species, Republic of Benin

## Abstract

**Background:**

Understanding the functional diversity of home gardens and their socio-ecological determinants is essential for mainstreaming these agroforestry practices into agrobiodiversity conservation strategies. This paper analyzed functional diversity of home gardens, identified the socio-ecological drivers of functions assigned to them, and assessed the agrobiodiversity benefits of home gardens functions.

**Methods:**

Using data on occurring species in home garden (HG) and functions assigned to each species by the gardeners, the study combined clustering and discriminant canonical analyses to explore the functional diversity of 360 home gardens in Benin, West Africa. Next, multinomial logistic models and chi-square tests were used to analyze the effect of socio-demographic characteristics of gardeners (age, gender, and education level), agro-ecological zones (humid, sub-humid, and semi-arid), and management regime (single and multiple managers) on the possession of a functional type of home gardens. Generalized linear models were used to assess the effect of the functions of home gardens and the determinant factor on their potential in conserving agrobiodiversity.

**Results:**

Seven functional groups of home gardens, four with specific functions (food, medicinal, or both food and medicinal) and three with multiple functions (more than two main functions), were found. Women owned most of home gardens with primarily food plant production purpose while men owned most of home gardens with primarily medicinal plant production purposes. Finding also showed that multifunctional home gardens had higher plant species diversity. Specifically, crops and crop wild relatives occurred mainly in home gardens with food function while wild plant species were mostly found in home gardens with mainly medicinal function.

**Conclusions:**

Home gardening is driven by functions beyond food production. These functions are mostly related to direct and extractive values of home gardens. Functions of home gardens were gendered, with women mostly involved in home food gardens, and contribute to maintenance of crops and crop wild relatives while men were mostly home medicinal gardeners and contribute to the maintenance of wild plant species in home gardens. Although multiple functional home gardens were related to higher plant diversity, there was no guarantee for long-term maintenance of plant species in home gardens.

## Background

Home gardens (HGs) are traditional farming systems, presumably one of the oldest land use system [1]. They occur in both rural and urban areas, temperate and tropical regions, low- and highland altitude, and low- and rich-income countries. Their benefits have been widely acknowledged in many domains including food and nutritional security [2–5], biodiversity conservation [6–12], economic hardship, poverty alleviation [13–16], ecosystem services provision [10, 17, 18], carbon sequestration [19–23], socio-cultural preservation [24–26], and in empowerment and social position of women [25, 27, 28].

During the last 12 years, African home gardens have received increasing interests from researchers. Although largely less documented in comparison to their Latin American and Asian counterparts, the research effort has spanned across their structure [29, 30], plant diversity and determinant factors [30–32], conservation benefits [6, 7, 33], uses and traditional knowledge associated to home garden flora [34], and factors determining their ownership and structure [30]. Still, many aspects of African home gardens remain blurred and call upon extensive research.

Home gardens are cultivation systems for both food and non-food production. Nevertheless, home gardens are mostly known for their food production function considered to be their basic function [4]. The different denominations associated to home gardens home food gardens, urban food gardens, domestic food gardens and kitchen garden [35–37] are evidences of the paramount importance attributed to food production function of home gardens in the available literature. However, based on the spectrum of home gardens’ ecosystem services [10, 17, 38–40] and the different uses reported to be associated to home gardens [7, 34, 41], the non-food productions (medicinal, ornamental, delimitation/protection, etc.) are also of importance especially in some geographical contexts. For instance in Benin, where the reported plant uses for non-food purposes compare the food ones [7, 42], it should be expected that home gardens are functionally diverse. Because food and health care are basic human needs, we predict that food and medicinal function will predominate the other functions.

Home gardens like other ecosystems have a wide range of material and non-material functions including provisioning (food, medicinal, fodder, etc.), cultural (social cohesion, recreational, symbolic, etc.), supporting (biodiversity), and regulating services (air purification, pollination, local climate regulation, maintenance of soil fertility, etc.) [43]. Because home gardens are most often established for provisioning functions [44, 45], this study therefore focused mainly on material uses of home gardens’ plants.

Questions that rises from such diversity of function are as follows: *what influences owner’s choice of a given functional type and how does functional diversity of home gardens affect conservation of specific groups of agro-biodiversity (namely crops, crop wild relatives and wild plant species)?* Understanding multifunctionality of home gardens requires accounting for the socio-economic and ecological processes occurring in these systems [30]. As such, accounting for function-based typology of home gardens could refine current understanding of factors shaping home garden ownership. Considering the effect of bio-cultural and economic factors as well as agro-ecological zone on plant diversity maintenance in home gardens [7, 29, 42, 46–48], this study predicts that the possession of a functional type of home garden is determined by both socio-economic conditions (gender, age, economic activity, education level) of gardeners and the agro-ecological zone they belong to. In particular, because of the labor division occurring in household in Africa, we predict women to be orientated towards home food-based gardens while men towards home non-food-based gardens. Young people are predicted to be orientated towards home food-based gardens due to their limited knowledge on medicinal plants. People involved in off-farm economic activities are expected to be more orientated towards food-based gardens as a way to leverage the volatility of food price and availability. With regards to agro-ecological zone, we predict high prevalence of food-based gardens in dryer zone as a strategy to guarantee fresh food and to cope with long food shortage uncertainties (leverage the volatility of food price availability).

Because of the difference in function of home gardens, these systems should also be expected to hold different potentials to maintain agrobiodiversity and specific groups of plant (crops, crop wild relatives, and wild plant species). We therefore predicted that multifunctional gardens will be associated to higher plant species richness. In addition, as functions of a given home garden are an expression of its structure mainly its species composition, we expected home gardens with specific function to harbor specific groups of plant. In particular, home gardens with food purpose are likely to maintain crops species and crop wild relatives than wild plant species, while home gardens with medicinal purpose are likely to maintain more wild plant species than crops species and their wild relatives.

This study analyzed African home gardens from a perspective of their functional characteristics. Specifically, this study aimed to (i) assess the functional diversity of home gardens, (ii) identify factors determining possession of a functional type of gardens, and (iii) determine the relationship between functional diversity of home gardens and conservation of specific groups of agrobiodiversity (crops, crop wild relatives, and wild plant species).

## Methods

### Study area

**Fig. 1:**
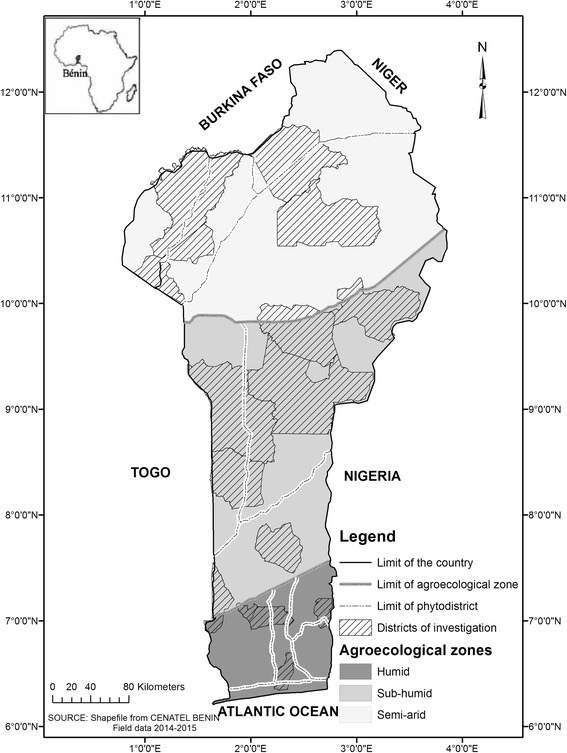
Agro-ecological zones, phyto-geographical districts, and administrative districts of investigation for a study of 360 home gardens in Benin

The study was carried out in Benin (Fig. 1). Benin is about 114.763 km^2^ [49], with three main agro-ecological zones (AEZs) ranging from humid to semi-arid which are distinguishable [50] with contrasting ecological (Table 1) and socio-economic characteristics. All the three zones were considered in this study. The resident population of about 10,008,749 inhabitants is unequally distributed [49], with 60% of the population concentrated in 20% of the territory [51]. The population is mainly young (more than 40% is under 15 years old) and slightly female-biased (51.2%) [49]. Thirty-three percent of the population has at least basic education (primary school or alphabetization in local languages) while the remaining part of the population can neither read nor write [51].

**Table 1:** Characteristics of the three agro-ecological zones of Benin

	Agro-ecological zones			
Parameters	Semi-arid	Semi-humid	Humid zone	Sources
Location	9°45′–12°25′ N	7°30′–9°45′ N	6°25′–7°30′ N	Sinsin et al. [52]
Rainfall (mm)	< 1000	900–1110	1200	Judex et al. [51]
Climate type	Dry tropical	Humid tropical	Humid tropical	Judex et al. [51]
Density of population^††^	33–49	51–162	191–8593	INSAE [49]
Days in growing season	90–100	180–270	270–365	Jahnke and Jahnke [50]

^††^ Inhabitant.km-2

Rainfall distribution in Benin shows two types of climates with a region of transition. In the south (humid and sub-humid zones), the climate is tropical humid with two rainfall maxima corresponding to two rainy seasons: March–July and September–November. The remaining months are dry [52, 53]. In the northern part (semi-arid zone), the climate is sudanian with one rainy season covering May to October and a long dry season covering November to May [52, 53].

Benin’s native vegetation is composed of fallows and small forest patches of less than 5 ha in the humid zone. The sub-humid zone consists of mosaics of woodlands, whereas the semi-arid zone consists of savannas and gallery forests with trees and shrubs slightly covering the ground [54].

Three major socio-linguistic groups are encountered in the Republic of Benin: Kwa, Gur, and Yoruboїd. Kwa is found mostly in the humid zone (i.e., mainly in Southern Benin but also in the central Benin) and includes the socio-linguistic groups Adja (and relatives), Mina (and relatives), Fon (and relatives), etc. [51]. Yoruboїd is found mainly in the sub-humid zone but also in south-eastern part of the humid zone and includes socio-linguistic groups Yoruba, Idaasha, and Nagot (and relatives) [51]. Gur is found in the semi-arid zone (Northern Benin) and includes socio-linguistic groups Bariba (and relatives), Gurma (and relatives), Lokpa (and relatives), Yom (and relatives), etc. [51].

### Sampling and data collection

Data considered in this paper are part of a larger data set collected in the framework of an ongoing research project on HGs in Benin [7, 30, 42]. The overall methodology developed for sampling and data collection consisted in four steps:

- Step 1. A rapid rural appraisal was carried out by a multidisciplinary team (agronomists, socio-economists, and ecologists), with agricultural extension services and local farmers’ organizations. A rapid rural appraisal is a participatory approach of diagnosis commonly used in field research on farming systems. It is considered as the critical first step in farming systems analysis [55, 56]. Here, it aimed at identifying villages and localities with high prevalence of home gardening practices. The main question during the interview was *where are important areas of home gardening in your region?*
- Step 2. In the previously identified villages and localities, an exploratory survey was conducted on 100 randomly selected informants in each AEZ. The survey intended to determine the proportion *p* of households with home gardens and consequently the sample size (*n*) in AEZ using the normal approximation of the binomial distribution [57].

1 $$ n=\frac{U_{1-\upalpha /2}^2\times p\ \left(1-p\right)}{d^{{}^2}} $$

In Eq. 1, ***U***_1 − ***α***/2_ the value of the normal random variable at a probability value of 1 − α/2. For the probability value of 0.975 (or α = 0.05), ***U***_1 − ***α***/2_ ≈ 1.96; *d* is the margin error of the estimation of any parameter value to be computed from the survey, and a value of 8% [30, 42] was chosen; and *p* is the proportion of households practicing home gardening. According to key informants (local agricultural extension services and local farmers’ organizations), *p* was estimated to be 20% for the humid zone, 31% for the sub-humid zone, and 34% for the semi-arid zone. As a result, 360 households were sampled: humid (96), sub-humid (129), and semi-arid (135) zones. In each AEZ, the project team visited the districts and villages selected as of high importance for home gardening.

- Step 3. Snowball technique [58] was used during visits of households with home gardens, to generate the list of households practicing home gardening. Three to five key informants (head of household with HG) were recruited and joined the team for this purpose. With their assistance, we walked around the villages and visited HGs. For each visited HG, we recorded the geographical coordinates using GPS Garmin 60, the name of the head of the household associated to the garden and took pictures. The list of HGs visited was established, and each HG was numbered.
- Step 4. From the established list of HGs (step 3), a random selection of participating households using the defined sample size (in step 2) was done based on a table of random numbers.

The 360 households were considered for both individual interviews and garden inventories. For the individual interview, we collected socio-economic and demographic information on garden owner. In this study, garden owner refers to a person (only one) recognized by the household and the community as responsible of the garden in terms of decision-making: location and design of the garden, selection and arrangement of species, cultural practices, and destination of outputs. This person may have installed the garden, inherited, or got it from a previous owner [59]. As garden may sometimes be managed by more than one household member, information on the management regime (single or multiple managers) was recorded for each garden, as well as the uses of maintained plant species.

Interviews generally lasted 60 to 90 min when the researcher could communicate directly with the informants, and more time (~ 120 min) whenever assistance of a translator or other relevant informant was required. Interviews were recorded primarily using a questionnaire. Additionally, a digital recorder was used to record all exchanges with informants (after informant consent) when necessary.

Socio-demographic characteristics of gardeners considered were age, gender, education level, ethnicity, and main economic activity. Following the age categorization used by [30, 42], 17.14% of the informants were young (age < 30), 63.20% were adult (30 < age < 60) and the remaining 19.66% were old people (age > 60). 44.38% of informants were female. Regarding education level, 36.24% were uneducated, 36.24% attended primary school or alphabetized classes and 27.52% attended secondary school or more.

Inventory data were collected between May 2014 and April 2015 with the assistance of local translators. HGs were visited in the rainy and the dry seasons to capture most of the variation in species composition. For each garden, an exhaustive inventory of plant species was carried out with the assistance of HG owner/tender. Weedy plant species, i.e., spontaneous plant species declared as unwanted in the gardens, were not inventoried. For all visited HGs, inventoried plants were identified at the species level and named following the botanical nomenclature of Lebrun and Stork [60]. Vouchers of plants that could not be clearly identified were collected and preserved following the Benin’s national herbarium guidelines for collecting herbarium specimens and sent to the national herbarium for identification by botanists.

Agrobiodiversity refers to the subset of natural biodiversity which includes the plant genetic resources used for food and agriculture [61] as well as wild edible and non-edible plants that are maintained in home gardens for different purposes. In this study, agrobiodiversity includes crops, crop wild relatives, and wild plants intentionally maintained or planted. Agrobiodiversity of home gardens was measured at the species level which is the last major taxonomic rank of the biological classification and considered as the most important by the International Code of Nomenclature for plants [62]. Even if the measurement of agrobiodiversity at infraspecific level (cultivars, races, sub-races, line, clone, ecotypes, etc.) might provide more precision, taking it into account is sometime challenging and may lead to bias as there are no rigid rules for the use of some of these terms [63], especially in the study area context.

Crop wild relatives (CWR), crops (CRP), and wild plant species (WPS) were identified using the following resources [52, 64, 65] and with assistance of the national herbarium and agricultural extension services. Finally, gardeners attributed uses for each plant species. All the uses were grouped into the main categories of uses in ethnobotany and usually reported in home gardens in Benin [7, 30, 42] (Table 2).

**Table 2:** Summary statistics of variables

Explanatory variables	Levels	Humid-region	Sub-humid region	Semi-arid region	Whole sample
Gender	Female	29	42	89	160
	Male	79	76	45	200
Age categories	Young	12	17	22	51
	Adult	63	66	89	212
	Old	27	31	28	86
Education level	Unedu	15	39	45	131
	PScho	31	55	44	130
	Second+	15	38	45	99
Management	Single	33	50	80	163
	Multiple	75	68	54	197
Dependent variables					
Agrobiodiversity	m ± se	10.29 ± 0.59	11.15 ± 0.59	9.25 ± 0.43	10.18 ± 0.31
Crops	m ± se	6.86 ± 0.38	7.42 ± 0.38	7.11 ± 0.32	7.14 ± 0.21
Crop wild relative	m ± se	0.56 ± 0.07	0.31 ± 0.04	0.5 ± 0.06	0.45 ± 0.03
Wild plant species	m ± se	2.86 ± 0.31	3.42 ± 0.33	1.64 ± 0.14	2.59 ± 0.16

*Unedu* uneducated people, *PScho* basic school (primary school or alphabetization), *Second+* secondary school or more, *Single* single manager, *Multiple* multiple manager, *m* mean, *se* standard error

### Statistical analysis

Five categories of HG plants use were considered: food, medicinal, ornamental, protection/delimitation, and miscellaneous (e.g., cultural, religious, insecticide, etc.). In this study, we considered that these uses define the function(s) assigned to the HG. Based on the usage(s) mentioned by the HG owner(s) or manager(s) for each species inventoried in his/her (their) HG, the proportion of species belonging to each of the above five categories of uses was calculated per HG. Species with multiple uses were considered simultaneously for the corresponding categories. For example, if a species was cited for food and medicinal uses, then it is counted for both use categories.

The matrix of 360 HGs by five columns corresponding to the 360 HGs and the above five use categories was submitted to a hierarchical clustering to define clusters of HGs with similar characteristics (uses patterns). A canonical discriminant analysis was then performed to assess how the uses categories discriminated the clusters of HGs. Mean value of each use category was calculated per cluster and plotted using radar chart. Therefore, it was possible to make a typology of HGs based on the most important uses of each cluster: types of HG were defined and named accordingly. Radar chart was used to illustrate the variation in function of home gardens. In the radar, each of the five use categories (function) was arranged radially around a central point. The value of each use category (function) is depicted by the node (anchor) on the spoke (axis). A line is drawn connecting the data values for each spoke. The seven clusters of home gardens were ranked on each of the five use categories (functions).

Because more than two types of HG were obtained, multinomial logistic model was used to assess the effect of AEZ (humid, sub-humid, semi-arid), age category (young, adult, old), gender (male, female), education level (not educated, basic education, secondary and plus), and management regime of HG (single versus multiple) on the occurrence of each functional type of HG. The full model was first fitted, and then backward elimination using likelihood ratio test was used to select the minimum adequate model. Because there was no significant interaction (*p* > 0.05), chi-square test was finally used to test the dependency between each factor and the occurrences of functional types of HGs. Barplots were used to illustrate the variation and describe the observed patterns.

To examine the link between diversity (overall species richness, crops, crop wild relative, and wild plants) in one hand and proportion of species in HGs devoted for each of the five use categories, pairwise Pearson correlation was used. Next, a Poisson GLM was used to compare the diversity of crops, crop wild relative, and wild species among functional types of home gardens. In this model, AEZ, management regime, and socio-economic factors (age category, gender, economic activity, and education level) were considered as additional predictors. Similar analysis was used for the overall species richness of HGs, with the difference that the GLM was based on the negative binomial error distribution to overcome over-dispersion in the data [66]. Backward elimination was used to select the minimum adequate model based on the value of AIC.

The hierarchical clustering was performed in SAS software version 9.2 while all other analyses were implemented in R software version 3.3.1 [67]. The canonical discriminant analysis was performed in package “Candisc” [68]. Before the canonical discriminant analysis, assumptions of multivariate normality and homogeneity of covariance matrices were checked with Mardia’s test in package *MVN* [69] and Box’s test in package *biotools* [70], respectively. The multinomial logistic model was implemented in package “epicalc” [71] and the GLM with negative binomial error distribution in package “MASS” [72].

## Results

### Functional typology of home gardens based on the spectrum of uses

**Fig. 2:**
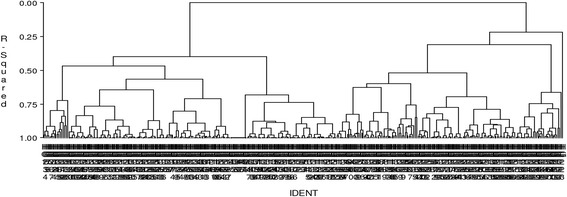
Hierarchical clustering of 360 home gardens based of the spectrum of their plant uses

The hierarchical clustering analysis applied on the spectrum of plant uses in 360 home gardens distinguished seven clusters of multiple gardens and one cluster with a single garden with conservation of 59.7% of the variation within the initial table (Fig. 2).

The canonical discriminant analysis examined differences in the five covariates (uses) between clusters of home gardens and indicated the relative contribution of each use to discrimination of home garden clusters. Three canonical discriminant axes were retained and accounted for 81.9% of between home gardens’ cluster variation. The likelihood ratio test showed that all the three canonical discriminant functions were significant at 1% significance level. The predominance (49.2%) of the first canonical discriminant function (Can1) and its relation with food, medicinal, and miscellaneous uses suggests that food, medicinal, and miscellaneous uses are prominent in home garden discrimination (Table 3).

**Table 3:** Correlation between each response variable and canonical discriminant axis

	Canonical discriminant axes		
	Can1	Can2	Can3
Variance explained	49.25%	17.69%	14.95%
LR-test stat approx	0.03	0.12	0.25
Pr(> *F*)	< 0.001	< 0.001	< 0.001
Uses	Correlation		
Food	*0.81*	− 0.04	0.01
Medicine	*− 0.78*	− 0.29	0.19
Ornament	− 0.23	*0.87*	0.03
Fence	− 0.15	− 0.19	*− 0.86*
Miscellaneous	*− 0.80*	0.04	− 0.27

**Fig. 3:**
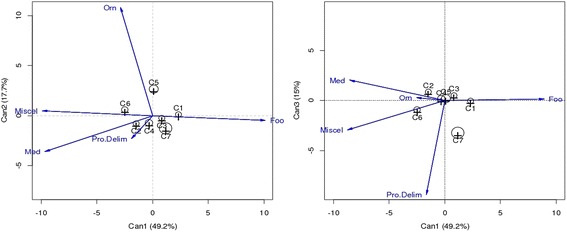
Projection of the variables (uses) and home gardens clusters in two systems of canonical discriminant axes: Can1-Can2 and Can1-Can3. Pro.Delim protection and delimitation

The first canonical axis (Can1) was correlated positively with food use and negatively with medicinal and miscellaneous uses. Therefore, food function was negatively correlated to non-food function (medicinal and miscellaneous). Projection of canonical scores for variables and clusters (Fig. 3a) indicated that home gardens of clusters 1, 7, and 3 were primarily food and significantly different from home gardens of clusters 6 and 2 which were primarily for non-food function and in lesser extend different from home gardens of clusters 4 and 5 which were for both food and non-food functions (medicinal and miscellaneous).

The second canonical axis (Can2) was correlated positively to ornamental use. Projection of canonical scores for uses and clusters (Fig. 3a) indicated that home gardens of cluster 5 was associated to ornamental function and significantly different from home gardens of clusters 1, 2, 3, 4, 6, and 7.

The third canonical axis (Can3) was negatively correlated to protection/delimitation use. Projection of canonical scores for uses and clusters (Fig. 3b) indicated that home gardens of cluster 7 were associated to protection/delimitation function and significantly different from home gardens’ clusters 1, 2, 3, 4, 5, and 6.

**Fig. 4:**
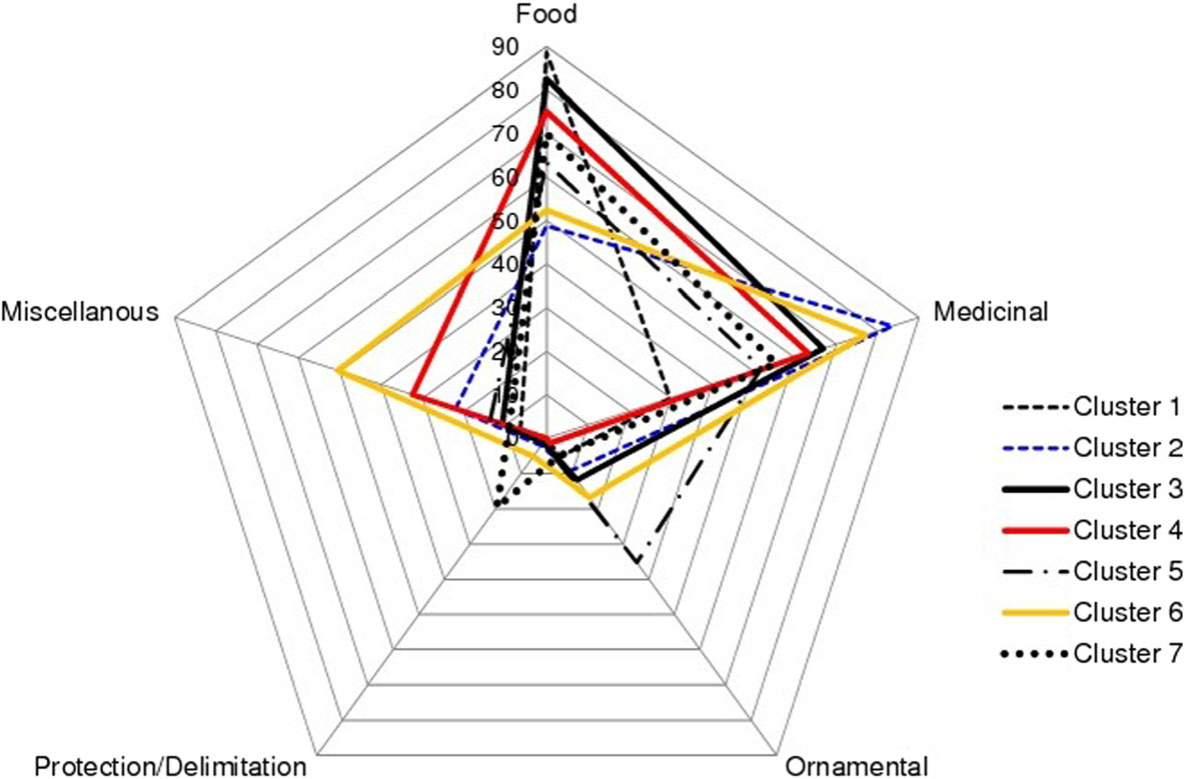
Average contribution of uses to the function of home gardens’ clusters

The average contribution of uses to the function of home gardens’ clusters (Fig. 4) indicated two main assemblages of home gardens: home gardens with specific function (one or two prominent functions) and home gardens with non-specific function (more than two prominent functions). Home gardens with specific function were primarily for food production and/or for medicinal plant production.

Within this assemblage, we distinguished:

- Primarily for food production (PF) home gardens comprising home gardens of cluster 1 with an average 88.64% ± 1.25 of their species devoted to food uses (Table 4).

**Table 4:** Summary statistics of functional categories of home gardens

		Uses				
Categories	Clusters	Food	Medicinal	Ornamental	Pro.Delim	Miscellaneous
PF	C1	88.64 ± 1.25	29.81 ± 1.97	5.329 ± 0.79	0.00	6.37 ± 0.96
PM	C2	48.79 ± 1.52	83.55 ± 1.57	9.292 ± 1.02	2.13 ± 0.5	22.02 ± 1.65
PFM	C3	82.51 ± 1.35	66.87 ± 1.49	11.82 ± 1.19	1.31 ± 0.34	10.69 ± 1.19
NSF	C4	75.13 ± 1.92	63.26 ± 2.71	1.38 ± 0.47	0.00	32.42 ± 1.87
NSF	C5	63.38 ± 2.81	51.88 ± 3.56	35.25 ± 2.90	1.38 ± 0.54	13.66 ± 1.85
NSF	C6	52.43 ± 2.40	77.07 ± 2.30	16.75 ± 1.32	5.21 ± 1.09	50.50 ± 2.02
PFM	C7	70.07 ± 3.64	55.44 ± 5.55	4.99 ± 1.66	19.55 ± 2.52	9.03 ± 2.99

*PF* primarily for food, *PM* primarily for medicine, *PFM* primarily for both food and medicine, *NSF* non-specific function, *Pro.Delim* protection and delimitation
- Primarily for medicinal plant production (PM) home gardens comprising home gardens of cluster 2 with an average of 83.55% ± 1.27 of their species devoted to medicinal purposes. (Table 4)
- Primarily for food and medicinal plant production (PFM) home gardens comprising home gardens of cluster 3 and in a lesser extent the gardens of cluster 7 with respectively an average of 82.51% ± 1.35 of their species devoted to food, 66.88% ± 1.49 of their species devoted to medicinal purposes and an average of 70.07% ± 3.64 of their species devoted to food, 55.44% ± 5.55 of their species devoted to medicinal purposes.

Home gardens with non-specific function were constantly for more than food and medicinal plant production and included for important part other uses as ornamental and protection/delimitation. Within this assemblage, we distinguished home gardens of cluster 4 for which in addition to food and medicinal uses, miscellaneous uses accounted for 32.42% ± 1.87 (Table 5); home gardens of cluster 5 for which in addition to food and medicinal uses, ornamental uses accounted for 35.25% ± 2.90 and home gardens of cluster 6 for which in addition to food and medicinal uses, miscellaneous uses accounted for 50.50% ± 2.02.

**Table 5:** Descriptive statistics of agrobiodiversity within the seven clusters of home gardens

Cluster	Crops species		Crops wild relatives		Wild species		Overall species richness	
	Range	m ± se	Range	m ± se	Range	m ± se	Range	m ± se
C1	1–18	6.77 ± 0.39	0–3	0.54 ± 0.09	0–6	0.84 ± 0.15	1–26	8.15 ± 0.49
C2	0–16	6.18 ± 0.42	0–2	0.32 ± 0.06	0–16	4.69 ± 0.45	1–30	11.19 ± 0.79
C3	2–19	8.72 ± 0.47	0–3	0.65 ± 0.09	0–6	1.58 ± 0.19	2–26	10.85 ± 0.64
C4	1–17	6.51 ± 0.50	0–2	0.33 ± 0.08	0–7	2.29 ± 0.26	2–22	9.13 ± 0.63
C5	1–19	8.00 ± 0.84	0–2	0.35 ± 0.12	0–7	2.26 ± 0.32	2–23	10.39 ± 1.08
C6	1–24	7.34 ± 0.61	0–2	0.32 ± 0.07	0–29	4.20 ± 0.55	2–52	11.86 ± 1.01
C7	2–13	6.12 ± 0.87	0–2	0.59 ± 0.19	0–5	1.88 ± 0.36	2–20	8.59 ± 1.11
Global	1–18	6.77 ± 0.39	0–3	0.44 ± 0.03	0–29	2.60 ± 0.16	1–52	10.14 ± 0.31
Prob.	< 0.001		< 0.001		< 0.001		< 0.001	

**Fig. 5:**
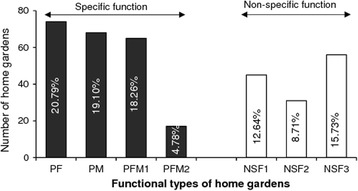
Occurrence of functional type of home gardens (*n* = 360) assemblages in Benin. PF primarily for food production, PM primarily for medicinal plant production, PFM primarily for food and medicinal plant production. Values in bracket are percentage in relation to the total number of sampled home gardens

With regard to the prevalence of home gardens assemblages, more than half of visited home gardens (59%) were with specific functions among which 21% were primarily for food, 19% were primarily for medicinal plant production and about quarter (24%) were primarily for both food and medicinal purposes (Fig. 5). The remaining home gardens (36%) were with non-specific functions (Fig. 5).

### Factors determining possession of a functional type of home garden and its plant diversity

**Fig. 6:**
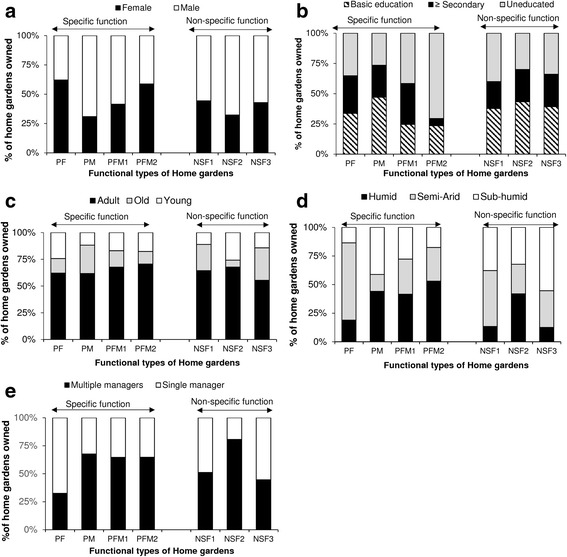
Relationship between possession of functional type of gardens, socio-demographic features of gardeners, ecological conditions the regime of management (**a**, **b**, **c**, **d**, **e**); PF primarily for food, PM primarily for medicine, PFM primarily for both food and medicine, NSF non-specific function

Gender was significantly associated to possession of functional type of garden (chi-square = 18.05, DF = 6; *p* value < 0.01). Women owned 62.16% of home gardens primarily for food production (Fig. 6a) and owned 45.12% gardens with both food and medicinal purposes (Fig. 6a). Men owned 69.12% of home gardens primarily for medicinal plant production, more than the half (54.88%) of for both food and medicinal home gardens and 59% of home gardens with non-specific function (Fig. 6a).

Education level and age of gardeners were not significantly associated to the possession of a functional type of home garden (chi-square = 19.19; DF = 12 for education level, chi-square = 17.71; DF = 12 for age of gardener, *p* value > 0.05). However, while having command in French did not determine the possession of a particular functional type of garden, people with at least a basic education owned most of home gardens with specific function respectively 64.86% of primarily for food home gardens, 73.53% of primarily for medicinal purpose home gardens and non-specific function/multifunction (Fig. 6b) and more than the half of primarily for both food and medicinal home gardens. Similarly, uneducated people and at large extent people with at most basic education owned most of home gardens (74.81%) with non-specific function (Fig. 6b). With regards to the age of the home gardeners, and regardless of the function, most of home gardens were owned by adult people. Primarily for medicinal and for both medicinal and food purposes, home gardens were almost exclusively owned by adult and old people, 88.24 and 82.93%, respectively (Fig. 6c).

The possession of functional types of garden was found to be significantly associated to agro-ecological zone (chi-square = 77.07; DF = 12 *p* value < 0.001). Home gardens with primarily for food production were mostly found in the semi-arid zone (67.57%) while home gardens primarily for medicinal purpose were mostly encountered in the sub-humid (41.17%) and humid zones (44.12%). Home gardens with primarily for both food and medicinal purposes were found everywhere but mostly in humid and sub-humid zones (Fig. 5d). Globally, 80.30% of home gardens with non-specific function were mostly found in sub-humid and semi-arid zones. However, those with high ornamental interest were mostly (41.93%) recorded in the humid zone (Fig. 5d).

With regards to the regime of management, possession of a functional type of home gardens was found to be significantly associated to the number of managers (chi-square = 33.64; DF = 6; *p* value < 0.001). Home gardens with primarily for food production were mostly found (67.57%) to have single manager while home gardens with primarily medicinal and/or food purposes were mostly found (66%) to be managed by at least two persons (Fig. 6e). Similarly, home gardens with non-specific functions were found to be fairly managed by at least two people, mainly when the gardens had high ornamental interest.

### Relationship between functional diversity of home gardens and agrobiodiversity

The analysis of the relationship between the whole plant diversity of home gardens and the spectrum of uses indicated positive and significant, although low correlation for medicinal use (*r* = 0.20, *p* value < 0.001), ornamental use (*r* = 0.19, *p* value < 0.001), and miscellaneous use (*r* = 0.17, *p* value < 0.01) (Table 6). Food and protection/delimitation uses were not significantly correlated to the global plant diversity (*r* = − 0.08 for food use, *r* = − 0.01 for protection/delimitation, *p* value > 0.05). Thus, home gardens with high interest for one or both of the following uses: medicinal, ornamental, and miscellaneous were likely to be more diversified than home gardens with high interest for food and protection/delimitation purposes.

**Table 6:** Correlation between the spectrum of plant uses, the global plant diversity, the diversity of crops, crops wild relatives, and wild plant species

Diversity level		Spectrum of plant uses				
		Foo	Med	Orn	Pro.Delim	Mis
Global	*r*	− 0.08	0.20	0.19	− 0.01	0.17
	*p* value	0.129	*< 0.001*	*< 0.001*	0.805	*0.001*
Crops	*r*	0.170	0.044	0.190	− 0.055	0.025
	*p* value	0.001	0.410	0.000	0.301	0.635
CWR	*r*	0.14	−0.09	0.03	− 0.01	− 0.08
	*p* value	*0.007*	0.092	0.530	0.880	0.140
Wild plant	*r*	−0.41	0.36	0.11	0.05	0.33
	*p* value	*0.000*	*< 0.001*	*0.038*	0.365	*< 0.001*

*Foo* food, *Med* medicine, *Orn* ornament, *Pro.Delim* protection and delimitation, *Mis* miscellaneous

The analysis of the relationship between specific groups of plant—crops, crop wild relatives, and wild plants—and the spectrum of uses (Table 6) indicated positive and significant correlation between food uses and diversity of crop wild relatives (*r* = 0.14, *p* value < 0.001). There was also positive and significant correlation between medicinal (*r* = 0.36, *p* value < 0.001), ornamental (*r* = 0.11, *p* value < 0.05), and miscellaneous (*r* = 0.33, *p* value < 0.001) uses and diversity of wild plant species but negative and significant correlation between food use (*r* = − 0.14, *p* value < 0.001) and diversity of wild plant species. Diversity of crops were not significantly correlated with ornamental (*r* = 0.07, *p* value > 0.05) and protection/delimitation (*r* = − 0.09, *p* value > 0.05) uses but was positively and significantly correlated to food uses (*r* = 0.27, *p* value < 0.001) while negatively correlated with medicinal (*r* = − 0.12, *p* value < 0.05) and miscellaneous (*r* = − 0.13, *p* value < 0.05) uses.

**Fig. 7:**
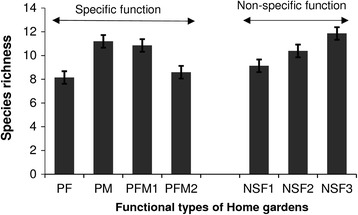
Relation between plant species richness and functional types of gardens

The generalized linear model describing the relationship between the plant diversity of gardens, and the clusters, the socio-economic factors, the agro-ecological zone, and the management regime indicated that only clusters had significant effect on the overall plant diversity of home gardens (*p* value < 0.001). Overall, home gardens with non-specific functions, i.e., multiple functional, were the richest (Fig. 7). Regarding home gardens with specific function, home gardens with primarily for medicinal and/or for food were also found to hold higher plant species richness (Fig. 7).

The generalized linear model describing the relationship between the sub groups of agrobiodiversity of gardens (crops, crop wild relatives, and wild plant) and the functional type of HG (with socio-economic characteristics, agro-ecological zones, and the management regime as additional predictors) indicated that:

- The diversity of crop plant species was similar among functional types of gardens. The average number of crops plant species was 2.87 ± 0.31. Among the additional predictors, both agro-ecological zone (*p* value < 0.01) and socio-demographic factors mainly gender (*p* value < 0.05) and education level (*p* value < 0.05) were significant. Higher diversity of crops was observed in the semi-arid zone (3.41 ± 0.20) and the lowest diversity observed in the humid (2.80 ± 0.180 and sub-humid (2.26 ± 0.15) zones. Higher diversity of crops was observed in home gardens owned by women (3.22 ± 0.17) while men had in average 2.56 ± 0.13 crops. Higher diversity of crops was recorded in home gardens of people with at least secondary school level (3.32 ± 0.25) while uneducated gardeners and gardeners with only basic education were found to have an average of respectively 2.65 ± 0.17 and 2.69 ± 0.19 crops.
- The diversity of crop wild relatives was similar among functional types of gardens. The average number of crop wild relatives was 0.44 ± 0.1. Among the additional predictors, only the agro-ecological zone was significant (*p* value < 0.05) with higher diversity of crop wild relatives observed in humid and semi-arid zones, respectively, an average (mean ± se) of 0.53 ± 0.07, 0.50 ± 0.06, and the lowest diversity occurring in sub-humid zone (0.31 ± 0.05).
- The diversity of wild plant species varied significantly (*p* value < 0.001) among functional types of gardens. Regarding functional type of HGs, high values of wild plant diversity occurred in home gardens with primarily for medicinal plant production (4.69 ± 0.45), in home gardens’ cluster 6 (4.20 ± 0.55), and in lesser extend in home gardens with non-specific function mainly cluster 4 (2.29 ± 0.26) and cluster 5 (2.26 ± 0.32). The lowest values of wild plant species were observed in home gardens with primarily for both food and medicinal purposes (1.88 ± 0.36 for cluster 7 (1.58 ± 0.19 for cluster 3) and in home gardens with primarily for food production (0.84 ± 0.15). Among the additional predictors, only the agro-ecological zone was significant (*p* value < 0.05) in determining the diversity of wild plant species. Higher values of wild plant species were observed in the sub-humid and humid zones with respectively 3.45 ± 0.33 and 2.84 ± 0.31 while the lowest value was observed in the semi-arid zone (1.65 ± 0.14).

## Discussion

This study examined the functional diversity of home gardens in Benin, assessed the effect of socio-economic conditions of gardeners, agro-ecological zones, and management regime on the possession of a functional type of home gardens. The study also took an additional step to examine how both functional type of home gardens and their determining factors could shape the diversity of specific groups of plants (wild plants, crop wild relatives, and crops).

The visited home gardens in Benin comprised of functional types of gardens from which four types (about two thirds of gardens) were of specific function (either food, medicinal, or both food and medicinal) while the three others types had multiple function (combining either protection/delimitation, ornamental or miscellaneous, and addition to food and medicinal uses). Possession of a functional type of gardens was found to be related to socio-economic profile of the gardeners, the management regime of the home-garden, and the agro-ecological zones. Finally, both functional type of home gardens and their determining factors were found to shape the agrobiodiversity of home gardens in particular wild plants, crop wild relatives, and crops.

The past classification of home gardens based on their functions [14, 30, 73–75] failed to answer a daunting question: Is food production (either for self-consumption or market-oriented) the only and main motivation of gardeners? Although food production is recognized as a basic function of home gardens [4], the motivation for home gardening is not always for mainly food production. Congruently with recent studies on home gardens in Benin [7, 42], findings revealed high prevalence of food and medicinal plants in gardens, confirming the importance of food production in gardening, and evidencing the key importance of medicinal plant in gardening systems in Benin. These two functions are therefore considered as the main motivations for home gardening in Benin. However, not all gardens are mainly for food and/or medicinal plant production. We also found gardens with relatively high proportion of plants devoted to ornamental, protection/delimitation, and miscellaneous (cultural, religious, etc.) purposes, which are associated to food and/ or medicinal plant production within home gardens. A relatively large part of home gardens in Benin (about 37%) were found under these functional types. Thus, even if existing, plant production for ornamental, protection/delimitation, and miscellaneous purposes is rarely the only motivation for home gardening in Benin. Overall, beyond the main motivation, most home gardens in Benin were associated to two, three, four, or more functions. This multi-functionality of home gardens is related to the large spectrum of ecosystem services expected from home gardens [10, 17, 38–40] and maintained by a continuous trade-off scheme occurring in home gardens [45]. Therefore, in the special case of Benin home gardens, we disagree with the statement that “the cooked is the kept” from Skarbø [44] and rather conclude that the useful is the kept in home gardens.

As gardens are managed for one or more desired functions, plant species are therefore proactively chosen to help each garden owner to satisfy his/her needs while coping with his/her social responsibilities. The function of garden is therefore influenced by both intrinsic characteristics of garden owner as well socio-environmental contexts. For instance, the functional types of gardens were found to be gendered. Home gardens with primarily for food production were generally owned by women while men were found to own most of home gardens with primarily for medicinal purposes. Similarly, home gardens with multiple functions including those with high interest for ornamental, protection/delimitation, and miscellaneous purposes were mostly owned by men. This specialization in home gardening is congruent with the traditional labor division and related social responsibilities at household level in African societies, where women are primarily committed to food issues while the protection of the household members including health cares and housing quality (protection/delimitation, ornament, etc.) are devoted to men. The latter responsibilities are associated to knowledge, wisdom, and wellness, and confer to men a higher social position. As women were also found to own home gardens for medicinal and/or food plant production, then home gardens are likely to increase the social status and the position of women as assumed by some researchers [25, 27, 28].

In another way and congruently to the previous observations of [7, 59] on the relationship between the agro-ecological zones and home garden plant composition, findings also revealed that the prevalence of functional types of garden varied across agro-ecological zones in Benin. Home gardens with primarily for food production were mostly found in the semi-arid zone where food security situation in Benin is alarming [76] and characterized by too long food shortage, and where large extends of farmlands are devoted to cotton production [77]. Home gardens with primarily for medicinal purpose were mostly encountered in sub-humid and humid zones. These zones were reported to host important traditional healers, medicinal plants’ markets [78, 79], and most of Benin’s forests [53]. Home gardens with primarily for both food and medicinal purposes and with more functions (ornamental, protection/delimitation, and miscellaneous purposes) were found everywhere but mostly in humid and semi-humid zones. In these latter zones, also known to be the most urbanized and under westernization [51, 80], households attempt to control fresh vegetables availability and price volatility by producing their own vegetables. These regions are also known to have typical gardens with almost a fencing configuration [81] to protect or delimit their homesteads. Home gardens with high prevalence of ornamental plant species were also mostly found in these regions under the westernization influence.

Regardless of the agro-ecological zone, home gardens with primarily for food or medicinal purposes had single manager while home gardens that combine food and/or medicinal plant production and ornamental, and/or protection/delimitation and/or miscellaneous purposes, were found mostly to be managed by at least two persons. The complex functional structure of gardens might suggest shared gardens as also observed in the Iberian Peninsula, Spain [82], wherein different household members value patches of lands around the homestead to comply with their specific needs and socio-economic responsibilities. Complex functional structure of gardens might also suggest a need for high labor investment, leading therefore to a labor division for handling garden management activities. This labor division in home gardens is related to garden location and design, crop type, specific plant species, etc. also observed by Howard (2004) in Latin America gardens.

Finally, results revealed that the function of home gardens (assumed to express the tender motivation) and controlling factors influence composition and diversity of plant species in home gardens. Indeed, findings showed that the multifunctional gardens had higher plant species richness meaning that the more there are functions in a home garden, the better it is for agrobiodiversity maintenance. At specific groups of the agrobiodiversity, wild plant species occurred mostly in home gardens with medicinal purpose either as primarily or associate production purpose, and in home gardens with multiple functions including those with ornamental, protection/delimitation and miscellaneous purposes. However, the composition and diversity of wild plant species in home gardens varied across agro-ecological zones, with higher richness recorded in humid and sub-humid zones. These observations suggest first that home gardens with medicinal function and/or with ornamental, protection/delimitation and miscellaneous plant production purposes were most appropriate for wild plant species maintenance and confirm the humid and sub-humid zones as hotspots for medicinal plants and sites to be selected for future conservation purposes. Regarding the crops and crop wild relatives, they occurred mainly in home gardens with food purpose either as primarily or associate production purpose, evidencing home gardens with food purpose as adequate for crops and crop wild relative maintenance in home gardens. Crops and their wild relatives were mostly recorded in humid and semi-arid zones, indicating these zones as hotspots for these resources at home gardens level [7] and representing then potential sites to be selected for future conservation purposes. Crops and their wild were mostly encountered in home gardens with primarily for food, generally owned by women, suggesting that women are key actors for crops and their wild relative maintenance in home gardens.

### Limitations of this work

This paper focused mainly on material uses of home gardens’ plants and to a lesser extent on non-material uses including ornamental uses (esthetic function) and miscellaneous uses (cultural, religious, etc.). The grouping of non-material uses into miscellaneous uses may have led to overlook the diversity of home gardens’ functions. However, the methods provide consistent results as regards the main uses of home gardens’ plants in Benin [34]. In another way, the agrobiodiversity was measured at the species taxonomical level, outlooking information at infraspecific level (e.g., variety, landrace, and ecotype). This limitation may have hindered a precise assessment of agrobiodiversity and the related uses and functions.

## Conclusions

Based on plant uses cited, HGs in Benin were found to be of specific or multiple functions. Overall, HGs either with specific or multiple functions were mostly related to consumption of primary goods (food, medicinal plants). Although HGs were known to have functions beyond provisioning services, gardeners through uses citations showed to be mostly interested in extractive values of gardens. Findings suggest also that the function of HGs was gendered with women mostly involved in specific function based garden and specifically home food gardens. Linking function to the composition and plant diversity of HG revealed that multi-functional HGs had higher plant diversity. However, there is no guarantee for long term maintenance of plant species in home gardens. It is important to notice that the motivation of gardener and consequently the function of home gardens may change with time. This change is a driver of home gardens dynamic. Although we still know less about factors affecting the dynamic of home gardens and their functions, their consequence on maintaining agrobiodiversity mainly crop wild relatives, crops, and wild plant species is of high concern for sustainable conservation.

## Abbreviations

AEZ: Agro-ecological zone
CRP: Crops
CWR: Crop wild relatives
HG: Home garden
HGs: Home gardens
PF: Primarily for food
PM: Primarily for medicine
PFM: Primarily for both food and medicine
